# Case report: Varicella-zoster virus infection triggering progressive encephalomyelitis with rigidity and myoclonus

**DOI:** 10.3389/fneur.2022.1042988

**Published:** 2022-11-29

**Authors:** Jing Yuan, Aihua Wang, Yunfeng Hou, Xuxu Xu

**Affiliations:** ^1^Department of Neurology, The First Affiliated Hospital of Shandong First Medical University and Shandong Provincial Qianfoshan Hospital, Jinan, China; ^2^Intensive Care Unit, The First Affiliated Hospital of Shandong First Medical University and Shandong Provincial Qianfoshan Hospital, Jinan, China

**Keywords:** progressive encephalomyelitis with rigidity and myoclonus, glycine receptor, varicella-zoster virus, herpes zoster, paroxysmal sympathetic hyperactivity

## Abstract

Progressive encephalomyelitis with rigidity and myoclonus (PERM) is a rare neurological disease of unknown etiology, and most patients with PERM are positive for anti-glycine receptor (GlyR) antibody. In this case study, we report a clinical case of a varicella-zoster virus-infected patient who developed anti-GlyR antibody-positive PERM. He initially suffered from herpes zoster and gradually developed symptoms of impaired brainstem functions including hoarse voice and dysphagia, accompanied by paroxysmal sympathetic hyperactivity. The patient also suffered from severe spasms, which were easily triggered by external stimuli. Glycine receptor antibodies were then found to be positive in serum and cerebrospinal fluid, and the diagnosis of PERM was confirmed. Methylprednisolone and gamma globulin treatments were given, and spasms were improved after treatment. Unfortunately, the patient's family insisted on automatic discharge and the patient passed away several days later.

## Introduction

Progressive encephalomyelitis with rigidity and myoclonus (PERM) is a rare autoimmune encephalitis syndrome characterized by muscle spasms, paroxysmal sympathetic hyperactivity (PSH), and brainstem and spinal cord symptoms (1). The pathogenesis of PERM is undetermined, but it is generally thought to be an autoimmune disease involving multiple receptor antibodies, ion channels, and related proteins, among which anti-glycine receptor (GlyR) antibody is the most common. In this case study, we present the case of a varicella-zoster virus-infected patient who developed PERM positive for autoantibodies against GlyR and responded to immunosuppressive therapy.

## Case report

A 75-year-old male patient (Han Chinese ethnicity) had headache and clusters of millet-sized blisters without fusion on the right side of the head and neck on February 24, 2022. The blisters were tight and shiny with surrounding redness, and the blister fluid was clear. The initial diagnosis was herpes zoster, and the patient was treated with 10 mg/kg of acyclovir every 12 h. On March 7, 2022, the patient's headache and herpes blisters were improved, so he stopped taking medicine. On March 12, 2022, the patient started to have left-sided headache, followed by herpes on the left side of the head and neck. He was diagnosed with recurrence of herpes zoster and was re-treated with acyclovir (10 mg/kg every 12 h). On March 16, 2022, the patient had hoarse voice and choking cough while keeping clear consciousness and movable limbs. He went to the Fifth People's Hospital of Jinan where he underwent clinical examinations on March 17, 2022. The CT scan of the lungs revealed pneumonia, and magnetic resonance brain imaging and CT imaging of the abdomen and pelvis did not demonstrate any significant abnormalities. An extensive biochemical assay revealed normal electrolytes, renal, thyroid, and parathyroid function, and negative serology for HIV, hepatitis B, and syphilis. The neurological examination showed the patient had clear consciousness and mental competence despite having decreased memory and impaired calculation, orientation, and judgment abilities. The patient could not speak clearly and fluently and had a choked cough when drinking water. Spasms were present with several minutes of paroxysmal painful muscle stiffness in the right upper limb, and the paroxysmal painful spasms were easily triggered by light touches and startling. The rest of the neurological examination was normal.

On March 19, 2022, the spasms frequently developed as a cluster accompanied by paroxysmal sympathetic hyperactivity (PSH), which was characterized by sinus tachycardia (110–160 beats/min), elevated systolic blood pressure (180–227 mmHg), tachypnea (30–50 breaths/min), and hypoxemia. An emergent electrocardiogram revealed that the patient had paroxysmal supraventricular tachycardia. The symptoms lasted for 3 min and were self-relieved. On March 21, 2022, the patient began with spasms of the right upper limb, followed by spasms involving all four limbs with loss of consciousness. Two minutes later, these symptoms were self-relieved. Due to his critical condition, the patient was transferred to the intensive care unit on the same day. His blood test results were unremarkable except for elevated serum levels of leukocytes (16.99 × 10^9/*L*^) and neutrophils (14.91 × 10^9/*L*^). A chest CT scan revealed manifestation of inflammation in both lungs, which was more aggravated than before. A lumbar puncture showed 22^*^10^6^ leukocytes/L (95% monocytes), and the rest of the biochemistry parameters were normal. At this point, the patient was diagnosed with central nervous system infection with high probabilities of varicella-zoster virus encephalitis, and the antibiotics treatment was switched to ceftriaxone sodium (2 g every 12 h) plus acyclovir (10 mg/kg every 12 h). Sedative and analgesic drugs as well as phenobarbital sodium were also given to control spasms. Recurrent spasms caused prolonged apnea, ultimately leading to a need for mechanical ventilatory support.

The patient's condition did not improve with these treatments, so he was transferred to the intensive care unit of the First Affiliated Hospital of Shandong First Medical University on March 29, 2022. The patient had coronary atherosclerotic heart disease in the past, could tolerate general physical activities, and had been diagnosed with hypertension for 3 months. The admission examination revealed that the patient had low and coarse breath sounds with scattered moist rales in the lower part of both the lungs. The patient was kept under sedation and mechanical ventilatory support. Laboratory examinations (performed on 29 March 2022) showed a leukocyte count of 11.48 × 10^9^/*L*, a neutrophil count of 9.96 × 10^9/*L*^, and a myoglobin count of 355.2 ng/mL. The rest of the biochemistry parameters were normal. The cerebrospinal fluid (CSF) opening pressure was 17 cmH_2_O, with 9 × 10^6^/L leukocytes/L and 43.4 mg IgG protein/L; normal protein and glucose levels; negative oligoclonal bands; and negative microscopy and culture for tuberculosis, fungi, and virology. Cardiac ultrasound and ultrasound of deep and superficial veins of bilateral lower extremities did not show any significant abnormalities.

**Figure 1:**
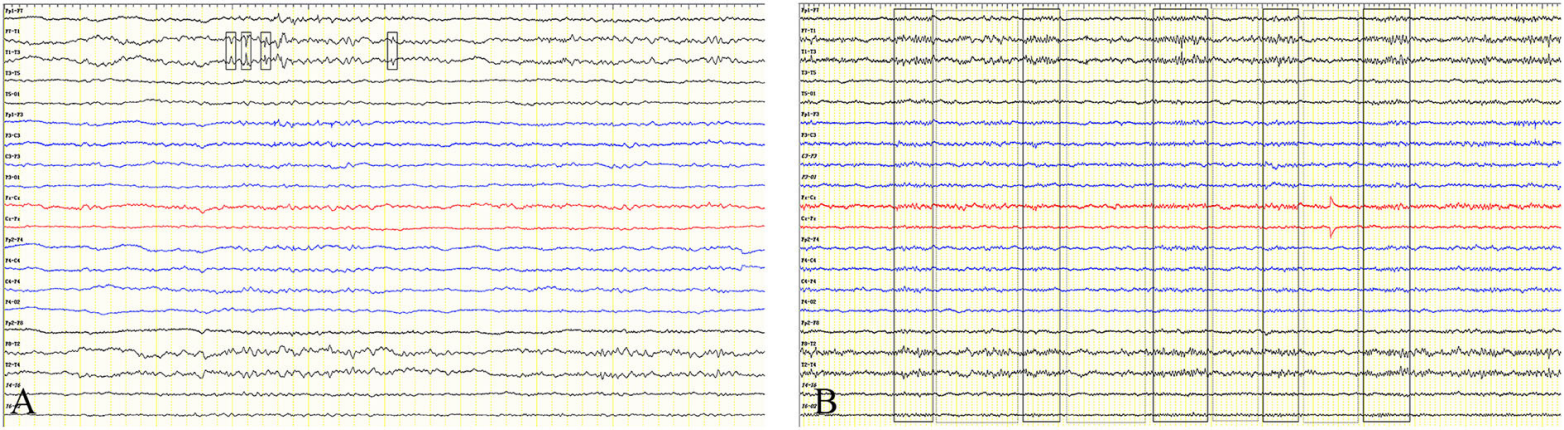
Electroencephalogram. The EEG showed **(A)** spike wave (thick solid rectangle) occurred in sphenoid bone and **(B)** slow waves (thick solid rectangle) interspersed with low amplitude of brain electrical activity (fine dotted rectangle) at regular intervals of 6–10 s.

**Figure 2:**
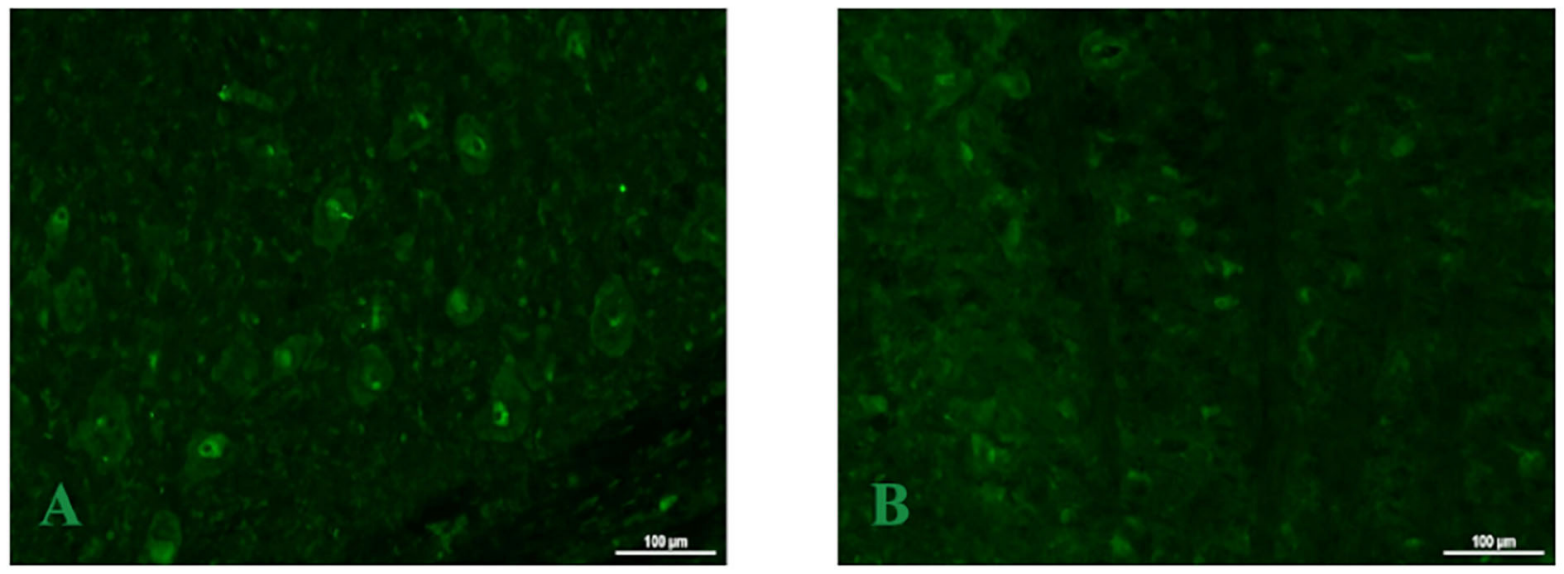
Blood and cerebrospinal fluid TBA screening. The TBA method was applied to incubate the patient's cerebrospinal fluid specimen with sections of cerebellar, hippocampal, cortical, and brainstem tissues from rats. Compared with the negative control **(B)**, the patient's cerebrospinal fluid **(A)** was present antibodies that might have the immune reactions with the rat brainstem tissues. The image shows specific fluorescent signals, indicating that there are positive cell membrane antibodies in brainstem neurons, whereas the patient's cerebrospinal fluid sample showed no fluorescence with rat cerebellar, hippocampal, and cortical tissue sections (data not shown).

**Figure 3:**
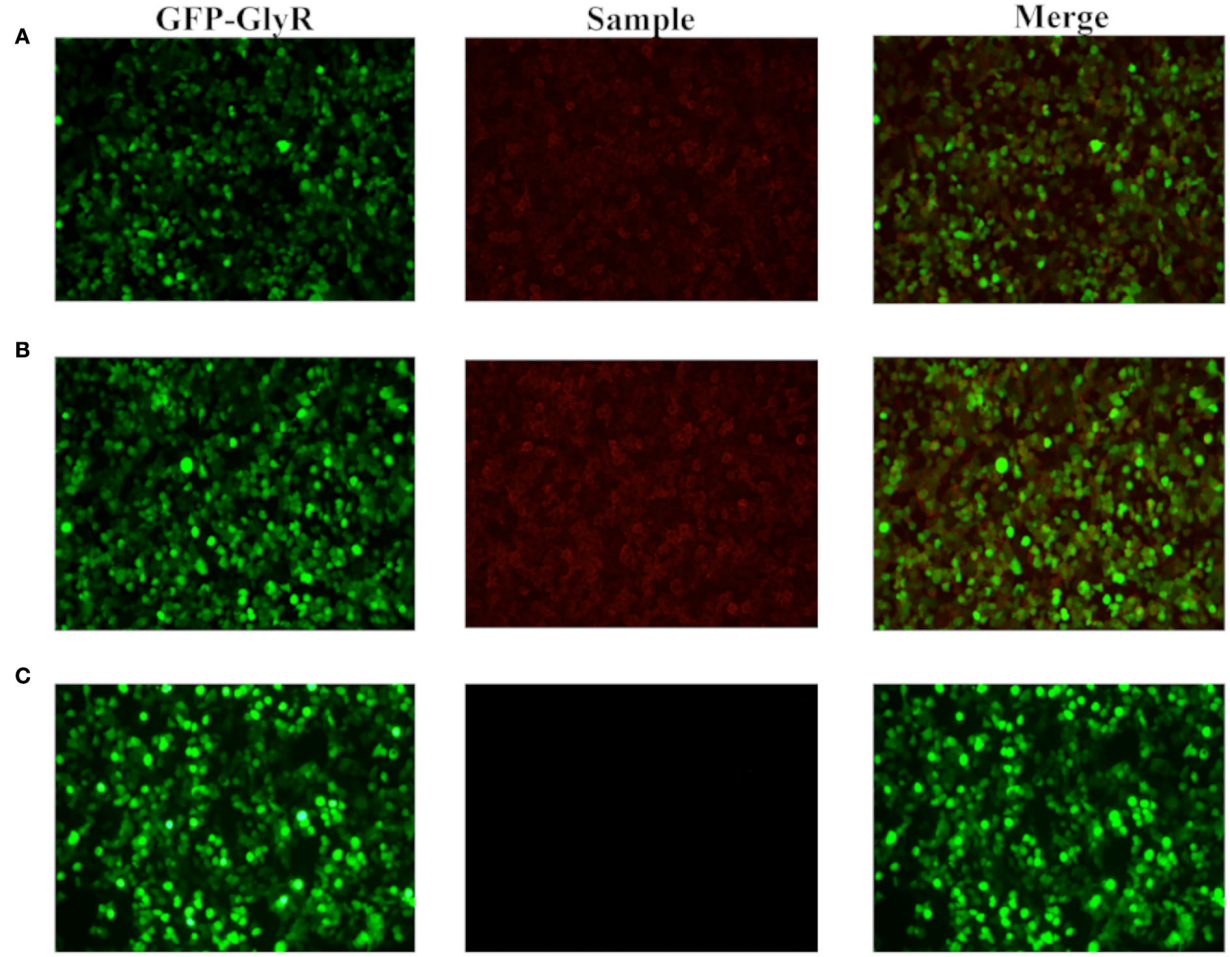
Blood and cerebrospinal fluid CBA screening. The CBA method was applied to incubate the patient's cerebrospinal fluid and serum (both diluted 1:100) with human embryonic kidney cell 293T (HEK293T) loaded with GlyR plasmids labeled with green fluorescent protein (GFP). The green fluorescence represents HEK293T cells containing the target antigen, the red fluorescence represents the fluorescent antibody in the specimen, and the positive means that the red fluorescence coincides with a specific part of the green fluorescent cell. Compared with the negative control **(C)**, there are autoantibodies that bind to GlyR expressed on the surface of HEK293T cells in the patient's cerebrospinal fluid **(A)** and serum **(B)**.

At that time, the patient was diagnosed with intracranial infection, so acyclovir (10 mg/kg every 8 h) was used. The sputum sucked from the patient was thick and purulent yellow, and the fungal 1–3-β-D-glucan quantitative test result of the sputum was 683.3 pg/mL. Therefore, the patient was suspected to have fungal infection; thus, voriconazole was given for antifungal treatment. Midazolam, remifentanil, phenobarbital sodium, and sodium valproate were added to further control the spasms. Despite an increased dosage of drugs, the spasms gradually worsened with increased frequency and could be triggered by phlegm suction, turning over, and backslap. Spasms began in the right upper limb, followed by muscle spasms in the left upper limbs, lower limbs, shoulders, neck, and back, which triggered a generalized tonic–clonic seizure (Supplementary Video 1). The electroencephalogram (EEG) showed slow waves (4–7 Hz) interspersed with low amplitude of brain electrical activity. Spike waves occurred in the sphenoid bone (Figure 1). Blood and cerebrospinal fluid tissue-based assay (TBA) screening showed weakly positive antibodies against cell membranes in the brainstem (Figure 2). No significant abnormalities were found in paraneoplastic antibodies (Hu, Yo, Ri, CV2, PNMA2, amphiphysin, recoverin, SOX1, titin, Zic4, GAD65, and Tr), and demyelinating antibodies (AQP4, MOG, MAP, and GFAP). Serum and cerebrospinal fluid cell-based assay (CBA) screening for autoimmune encephalitis antibodies showed that IgG antibodies against glycine receptor (GlyR) was positive at 1:100 in both serum and cerebrospinal fluid (Figure 3), while the remaining proteins or receptor antibodies (NMDAR, LGI1, CASPR2, GABABR, AMPAR1, AMPAR2, IgLON5, DPPX, GAD65, and mGluR5, D2R) were all negative.

**Figure 4:**
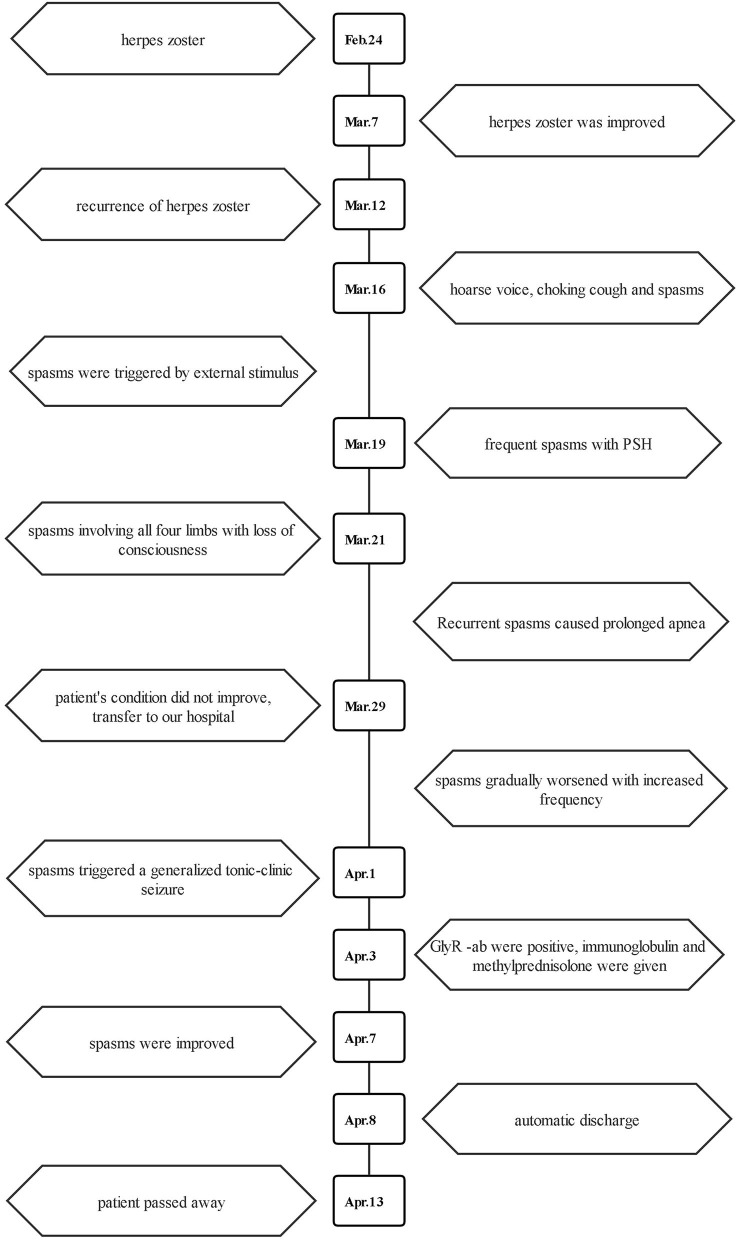
A schematic showing the timeline of the patient's conditions.

Putting the patient's clinical symptoms and auxiliary test results together (Figure 4), a diagnosis of anti-GlyR antibody-positive PERM induced by varicella-zoster virus was made. The patient was then given 0.4 g/kg/d immunoglobulin treatment for 5 days. Considering fungal infection, he was also temporarily treated with methylprednisolone (120 mg/d). After the 5-day treatment, the spasms were significantly less frequent than before. However, the patient's family insisted on automatic discharge despite continuous persuasion from the medical staff and actively helping in raising funds for the hospitalization on April 8, 2022. The patient then passed away on April 13, 2022.

## Discussion

The patient had typical clinical manifestations of PERM disease as follows: (1) Muscle spasms: The patient suffered from spasms of the right upper limb, which were easily triggered by light touches, startling, phlegm suction, turning over, and backslap. The electroencephalogram did not show typical spike and slow waves during interictal periods. We proposed several possible mechanisms: (i) The use of antiepileptic drugs interfered with the result of EEG. (ii) The EEG in patients of PREM could demonstrate no significant abnormalities, which was consistent with that of some previous studies (2, 3). (2) PSH: The patient had manifestations of sympathetic nerve excitation, such as sinus tachycardia, elevated systolic blood pressure, tachypnea, and hypoxemia. The symptoms of PSH accelerated the deterioration of the patient's conditions. (3) Auxiliary examination results: The results of patient's cerebrospinal fluid test showed immuno-inflammatory changes, and high titers of serum and cerebrospinal fluid anti-GlyR antibodies were detected. The patient's medical history and auxiliary examination results ruled out the occurrence of trauma, poisoning, tumor, and paraneoplastic and demyelinating diseases. Putting the abovementioned pieces of evidence together, we determined that the patient met the diagnosis standard of anti-GlyR antibody-positive PERM.

The pathogenesis of PERM is unclear, but it is currently considered to be a multiple-antibody-mediated autoimmune disease. Glutamate decarboxylase (GAD) and GlyR antibodies are found in most patients with PERM (4). Amphoteric proteins, dipeptidyl peptidase-like protein 6 antibodies, voltage-gated potassium channel complexes, and leucine-rich glioma-inactivating protein-1 have also been reported to be present in patients with PERM (1, 5–7). In the current case study, the patient had typical clinical manifestations of PERM, but it was unique in that varicella-zoster virus infection preceded the disease. Although there was a lack of etiologic evidence, the typical clinical symptoms and the effectiveness of antiviral therapy suggested varicella-zoster virus infection. This has revealed a potentially new pathogenetic mechanism by which varicella-zoster virus infection could induce an immune response against GlyR. Carvajal-Gonzalez et al. have confirmed at least 13 patients had autoimmune/inflammatory events preceding the infection (3), which implies that the pathogenesis of PERM may be attributable to an imbalance of immune homeostasis due to immunity or infection. Several studies have reported that viral infection is one of the mechanisms for autoimmune pathogenesis. For example, herpes zoster virus and EB virus can cause GD1a and GM1 antibody-associated Guillain–Barre syndrome (8, 9). Herpes simplex virus can induce N-methyl-D-aspartate receptor (NMDAR) antibody-associated encephalitis (10). A recent report showed that West Nile virus can trigger anti-GlyR antibody-positive PERM (11), suggesting that viral infection may indeed be one of the pathogenesis mechanisms of PERM. Varicella-zoster virus infection has been reported to cause NMDAR antibody-positive autoimmune encephalitis (12, 13). A study between varicella-zoster virus infection and anti-GlyR antibody immune response has not been reported. After reviewing the literature and careful examination of the current case, we propose several possible mechanisms: (1) Varicella-zoster virus can infect the brainstem, where the GlyR antigen is highly expressed (14), damage the brainstem structure, and lead to exposure and modification of GlyR antigen epitopes which become targets of autoimmune responses. (2) Varicella-zoster virus infection leads to the activation of T and B cells, which in turn secrete a large number of inflammatory cytokines that may cross the blood–cerebrospinal fluid barrier and cause an immune response in the central nervous system (15, 16), where the GlyR antigenic determinants are exposed and released as autoimmune targets. (3) Molecular mimic: Sequence or structural similarities between microbial and autoantigen epitopes might cause the immunological reaction to evolve to autoimmunity, which needs subsequent experiments to prove.

GlyR antibody-associated PERM syndrome is a rare disorder that can be life-threatening, if not treated. Most cases show improvement with corticosteroids, immunoglobulin, and plasmapheresis, and patients generally have a relatively good outcome (17). In our patient, the treatment was ineffective due to the delayed immunomodulatory treatments. In addition, high-dose hormone shock treatment was not given because of fungal infection. We planned to give the patient 500 mg/d methylprednisolone treatment after the pulmonary infection was relieved. Even though we applied only a small dose of hormone therapy to the patient, his spasms improved significantly. The patient in this case had a long disease course, diverse clinical manifestations, and many sudden changes in health conditions. By reviewing our experiences in this case, we should expand our understanding of immune diseases of the central nervous system and increase screening of antibodies for autoimmune diseases, paraneoplastic diseases, and demyelinating disease to prevent missed diagnosis or misdiagnosis and to achieve early diagnosis and effective treatment. The ultimate goal is to cure patients and reduce the emotional and financial burdens on patients and their family members.

## Data availability statement

The datasets presented in this article are not readily available because of ethical and privacy restrictions. Requests to access the datasets should be directed to the corresponding author.

## Ethics statement

Written informed consent was obtained from the individual(s) and minor(s)' legal guardian/next of kin, for the publication of any potentially identifiable images or data included in this article.

## Author contributions

JY and XX conceived the idea and wrote the manuscript. JY, XX, and AW performed the revision of the current literature and mainly gave to the draft an obstetrical perspective. YH and XX corrected and supervised the work. All authors approved the final version of the draft.

## Funding

This work was supported by the Youth Fund Project of National Natural Science Foundation of China (82001285) and the Natural Science Foundation of Shandong Province (ZR2020QH113) to XX.

## Conflict of interest

The authors declare that the research was conducted in the absence of any commercial or financial relationships that could be construed as a potential conflict of interest.

## Publisher's note

All claims expressed in this article are solely those of the authors and do not necessarily represent those of their affiliated organizations, or those of the publisher, the editors and the reviewers. Any product that may be evaluated in this article, or claim that may be made by its manufacturer, is not guaranteed or endorsed by the publisher.

## Supplementary material

Supplementary Video 1 This video was recorded on April 1, 2020, and the patient has been transferred to the ICU of our hospital for treatment. The patient was kept under sedation and mechanical ventilatory support. Spasms began in the right upper limb, followed by muscle spasms in the left upper limbs, lower limbs, shoulders, neck, and back, which triggered a generalized tonic–clonic seizure.

## References

[1] Shugaiv E Leite MI Sehitoglu E Woodhall M Çavuş F Waters P. Progressive encephalomyelitis with rigidity and myoclonus: a syndrome with diverse clinical features and antibody responses. Eur Neurol. (2013) 69:257–62. 10.1159/000342237 23429048

[2] Swayne A Tjoa L Broadley S Dionisio S Gillis D Jacobson L. Antiglycine receptor antibody related disease: a case series and literature review. Eur J Neurol. (2018) 25:1290–8. 10.1111/ene.13721 29904974 PMC6282944

[3] Carvajal-Gonzalez A Leite MI Waters P Woodhall M Coutinho E Balint B. Glycine receptor antibodies in PERM and related syndromes: characteristics, clinical features and outcomes. Brain. (2014) 137:2178–92. 10.1093/brain/awu142 24951641 PMC4107739

[4] Yao Q Fu M Ren L Lin C Cao L. Inspiratory laryngeal stridor as the main feature of progressive encephalomyelitis with rigidity and myoclonus: a case report and literature review. BMC Neurol. (2022) 22:42. 10.1186/s12883-022-02555-y 35090404 PMC8796497

[5] Balint B Jarius S Nagel S Haberkorn U Probst C Blöcker IM. Progressive encephalomyelitis with rigidity and myoclonus: a new variant with DPPX antibodies. Neurology. (2014) 82:1521–8. 10.1212/WNL.0000000000000372 24696508

[6] Ishii A Hayashi A Ohkoshi N Matsuno S Shoji S. Progressive encephalomyelitis with rigidity associated with anti-amphiphysin antibodies. J Neurol Neurosurg Psychiatry. (2004) 75:661–2. 10.1136/jnnp.2003.010504 15026525 PMC1739000

[7] Crisp SJ Balint B Vincent A. Redefining progressive encephalomyelitis with rigidity and myoclonus after the discovery of antibodies to glycine receptors. Curr Opin Neurol. (2017) 30:310–6. 10.1097/WCO.0000000000000450 28306573

[8] Hao Y Wang W Jacobs BC Qiao B Chen M Liu D. Antecedent infections in Guillain-Barre syndrome: a single-center, prospective study. Ann Clin Transl Neurol. (2019) 6:2510–7. 10.1002/acn3.50946 31714025 PMC6917331

[9] Shahrizaila N Lehmann HC Kuwabara S. Guillain-Barre syndrome. Lancet. (2021) 397:1214–28. 10.1016/S0140-6736(21)00517-1 33647239

[10] Armangue T Spatola M Vlagea A Mattozzi S Cárceles-Cordon M Martinez-Heras E. Frequency, symptoms, risk factors, and outcomes of autoimmune encephalitis after herpes simplex encephalitis: a prospective observational study and retrospective analysis. Lancet Neurol. (2018) 17:760–72. 10.1016/S1474-4422(18)30244-8 30049614 PMC6128696

[11] Karagianni P Alexopoulos H Sourdi A Papadimitriou D Dimitrakopoulos AN Moutsopoulos HM. West Nile Virus infection triggering autoimmune encephalitis: pathophysiological and therapeutic implications. Clin Immunol. (2019) 207:97–9. 10.1016/j.clim.2019.07.007 31454696

[12] Granerod J Ambrose HE Davies NW Clewley JP Walsh AL Morgan D. Causes of encephalitis and differences in their clinical presentations in England: a multicentre, population-based prospective study. Lancet Infect Dis. (2010) 10:835–44. 10.1016/S1473-3099(10)70222-X 20952256

[13] Schabitz WR Rogalewski A Hagemeister C Bien CG. VZV brainstem encephalitis triggers NMDA receptor immunoreaction. Neurology. (2014) 83:2309–11. 10.1212/WNL.0000000000001072 25378669

[14] Fujino Y Shiga K Hori M Tamura A Iizuka T. Case report: dexmedetomidine for intractable clusters of myoclonic jerks and paroxysmal sympathetic hyperactivity in progressive encephalomyelitis with rigidity and myoclonus. Front Neurol. (2021) 12:703050. 10.3389/fneur.2021.703050 34322087 PMC8311021

[15] Fujimura T Yamanashi R Masuzawa M Fujita Y Katsuoka K Nishiyama S. Conversion of the CD4+ T cell profile from T(H2)-dominant type to T(H1)-dominant type after varicella-zoster virus infection in atopic dermatitis. J Allergy Clin Immunol. (1997) 100:274–82. 10.1016/S0091-6749(97)70236-7 9275152

[16] Traina-Dorge V Sanford R James S Doyle-Meyers LA de Haro E Wellish M. Robust pro-inflammatory and lesser anti-inflammatory immune responses during primary simian varicella virus infection and reactivation in rhesus macaques. J Neurovirol. (2014) 20:526–30. 10.1007/s13365-014-0274-2 25139181 PMC4394654

[17] Chang A Lin KY Chuang KJ Waters P Irani S Mgbachi V. Progressive encephalomyelitis with rigidity: a Taiwanese case and review of literature. Clin Neurol Neurosurg. (2021) 208:106807. 10.1016/j.clineuro.2021.106807 34325335

